# Mother tongue? Muttersprache? Lingua mater? Not so fast!

**DOI:** 10.1093/nsr/nwy114

**Published:** 2018-10-08

**Authors:** Dan Graur

**Affiliations:** 1 Department of Biology and Biochemistry, University of Houston, USA; 2 Reviewer of NSR

In most languages, the arterial language, i.e. the language that a person has been exposed to from birth or within the critical period of language learning, is referred to as the ‘mother tongue’. Whether or not this phrase is factually correct has been a matter of debate for a long time.

One way to tackle this problem is to use genetic markers, and look at their concordance or discordance with linguistic geographical distributions and phylogenetic relationships.

To the best of my knowledge, the first study into the relationship between a genetic marker and a linguistic marker was published by C. D. Darlington [1]. He used the frequency of ABO blood groups as a genetic marker, and the existence or absence of the TH sound in a language as a linguistic marker. The sound map was found to agree with the ABO blood-group frequency contour map in Europe. Interestingly, Darlington believed that particular languages are determined by particular genetic makeups, rather than covarying with human migrations.

The first modern studies into genetic markers and the evolution of languages are considered to be those conducted in the middle of the 1960s by Luigi Luca Cavalli-Sforza and colleagues (e.g. Modiano *et al*. [2]). However, these studies were limited by data availability and relied mostly on blood groups.

So far, the ‘mother tongue’ hypothesis has received extremely limited support in the Austronesian-speaking populations [3] and the South-American native populations [4]. It is possible that these results are artifacts due to several factors: (i) genetic variation in these populations is less structured than that found elsewhere; (ii) the populations have minuscule effective population sizes, i.e. stochastic processes may rapidly destroy signatures of past migration events; (iii) gene flow is limited; and (iv) migration generally occurs within linguistic groups.

In the late 1990s, Laurent Excoffier and colleagues started looking at linguistic correlations with Y-linked genetic markers versus mitochondrial genetic markers with the aim of distinguishing between paternal and maternal effects on language parentage [5]. In African and European samples, they discovered that both Y-linked and mitochondrial variability were better predictors of linguistic distances than geographic distances. Interestingly, by keeping the mitochondrial variability constant, the Y-linked variability significantly explained 35% of the variation in linguistic distances, while by keeping the Y-linked variability constant, the mitochondrial variability explained only 5% of the variation in linguistic distances. Because languages seems to be passed on from generation to generation along the paternal lineage, Poloni et al. [5] concluded their paper with the recommendation to replace ‘mother tongue’ with the concept of ‘father tongue’.

Given the fact that most children acquire their arterial language from their mothers, the ‘father tongue hypothesis’ raises the problem of mothers teaching their children the language of the father even if they are not native speakers of this language. To resolve this problem, Zhang *et al.* (2018) distinguished two linguistic components and analyzed their relationships to genetic distances separately: lexical and phonemic distances [6]. Briefly, lexical distances quantify the percentage of cognates (worlds sharing the same origin) between two languages. Phonemic distances measure the percentage of shared perceptually distinct units of sound. Their conclusion was that while genetic and linguistic distances are significantly correlated with each other as well as with geographical distances, when controlling for geographic factors, only the correlation between the paternal distances and the lexical distances, and the correlation between the maternal distances and the phonemic distances remained significant.

The words are your father's; the way you say these words are your mother's.
